# Unique EEG signature of atypical absence seizures in SYNGAP1‐related developmental and epileptic encephalopathy

**DOI:** 10.1002/epd2.70295

**Published:** 2026-06-08

**Authors:** Rainier Mark Loidor L. Rapal, Joshua Chang, Douglas Nordli

**Affiliations:** ^1^ Department of Pediatric Neurology University of Chicago Chicago Illinois USA

We report a case of a 13‐year‐old girl with SYNGAP1‐associated developmental and epileptic encephalopathy. Seizures began at age 2, with atonic and atypical absence semiology. Brain MRI was normal. She has drug‐resistant epilepsy despite lamotrigine and topiramate.

Her interictal EEG showed diffuse slowing and disorganization, with poor anterior–posterior gradient (Figure 1). There were frequent bursts of diffusely distributed and pleomorphic slow spike and wave interictal discharges. Ictal EEG during atypical absence seizures showed slow eyelid closure followed by occipitally predominant spike–wave discharges (Figure 2). This crescendoed into a generalized 2 Hz–2.5 Hz spike and polyspike and wave pattern, ending with diffuse slowing.

**FIGURE 1:**
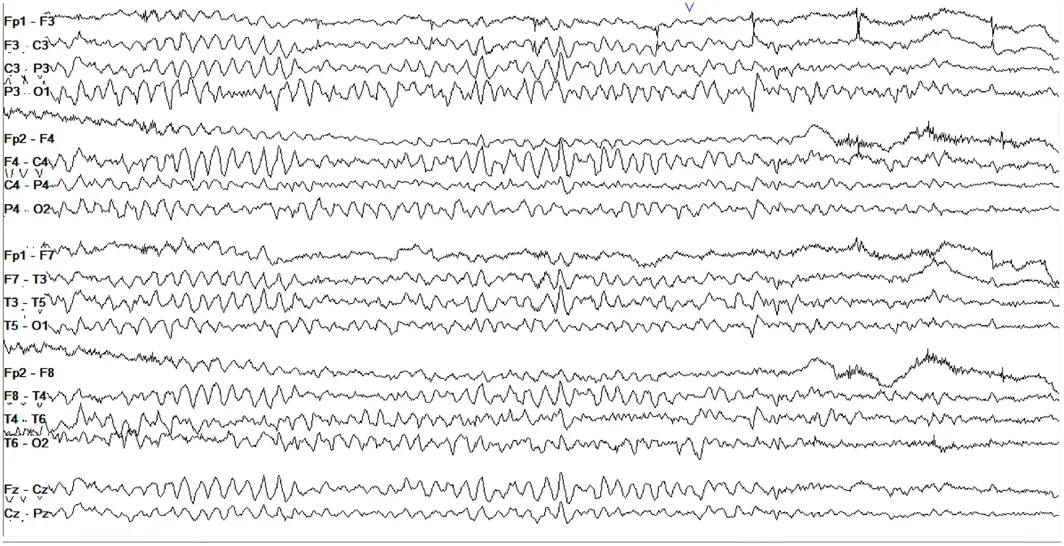
Interictal EEG background demonstrating diffuse slowing and disorganization with poor AP gradient.

**FIGURE 2:**
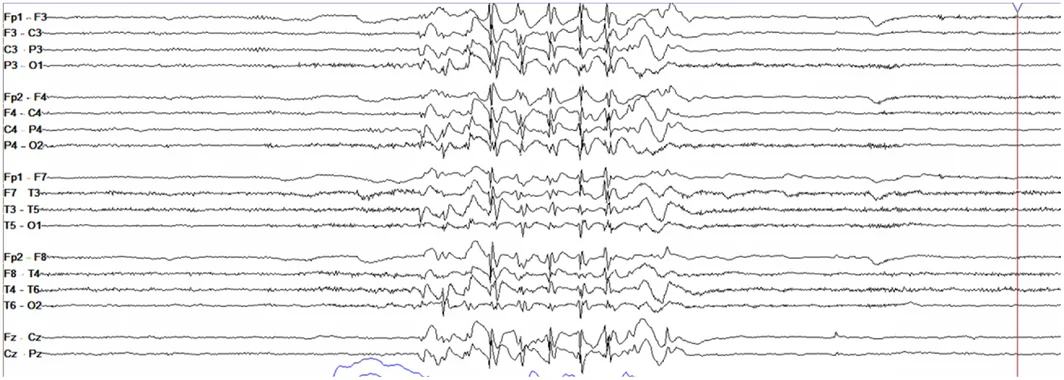
Atypical absence ictal EEG demonstrating findings of slow eyelid closure followed by occipital‐predominant discharges that crescendo into a generalized rhythmic spike‐and‐wave pattern.

SYNGAP1‐DEE is a monogenic epilepsy with known EEG features of background slowing and generalized or multifocal discharges.^1^, ^2^ A unique feature is atypical absence seizures resembling Jeavons syndrome with slow eyelid closure followed by occipitally‐predominant discharges that crescendo into a generalized pattern, emphasizing the need for neurologists to consider both conditions diagnostically.

## AUTHOR CONTRIBUTIONS

Conception and design: Rainier Mark Loidor L. Rapal, Joshua Chang and Douglas Nordli. Data Collection: Rainier Mark Loidor L. Rapal and Joshua Chang. Data analysis and interpretation: Rainier Mark Loidor L. Rapal, Joshua Chang and Douglas Nordli. Manuscript drafting: Rainier Mark Loidor L. Rapal and Douglas Nordli. Figures preparation: Joshua Chang and Douglas Nordli. Critical revision of manuscript: Rainier Mark Loidor L. Rapal, Joshua Chang and Douglas Nordli.

## FUNDING INFORMATION

No funding was received.

## CONFLICT OF INTEREST STATEMENT

No conflict of interest to be declared.

## CONSENT

Informed consent was obtained from the parent of the patient presented prior to beginning this work.

**Test yourself**

1. What is the most characteristic ictal EEG pattern in SYNGAP1‐related atypical absence seizures?
  - Frontally predominant 3 Hz spike–wave.
  - Occipital‐predominant spike–wave with posterior lead‐in and slow eyelid closure.
  - Centrotemporal spikes evolving to generalized discharges.
  - Multifocal spikes without generalization.
2. In SYNGAP1 developmental and epileptic encephalopathy, what interictal EEG feature is commonly described and correlates with cognitive outcomes?
  - Normal posterior dominant rhythm >8 Hz.
  - Background slowing with 6–7 Hz PDR and OIRDA maturing to biparietal theta.
  - Continuous generalized spike–wave in sleep.
  - Focal temporal slowing only.
3. Which semiology feature distinguishes SYNGAP1‐DEE absences from typical childhood absence epilepsy?
  - Brief staring without motor signs.
  - Hyperventilation‐induced 3 Hz absences only.
  - Slow eyelid closure, eye‐rolling, atonic drops, often fever/heat triggered.
  - Myoclonic jerks preceding loss of awareness.

*Answers may be found in the* *Supporting information*.

## Supporting information


**Data S1**.

**Data S2**.

## Data Availability

The data that support the findings of this study are available on request from the corresponding author. The data are not publicly available due to privacy or ethical restrictions.

## References

[1] Vlaskamp DRM , Shaw BJ , Burgess R , Mei D , Montomoli M , Xie H , et al. SYNGAP1 encephalopathy. A distinctive generalized developmental and epileptic encephalopathy. Neurology. 2019;92(2):e96–e107. 10.1212/WNL.0000000000006729 PMC6340340 30541864

[2] Ribeiro‐Constante J , Tristán‐Noguero A , Martínez Calvo FF , Ibañez‐Mico S , Peña Segura JL , Ramos‐Fernández JM , et al. Developmental outcome of electroencephalographic findings in SYNGAP1 encephalopathy. Front Cell Dev Biol. 2024;12:1321282. 10.3389/fcell.2024.1321282 PMC10948473 38505260

