# Supplementary material for: Unique EEG signature of atypical absence seizures in SYNGAP1‐related developmental and epileptic encephalopathy
Source: Epileptic Disord. 2026 Jun 8;28(5):1756–8. doi: 10.1002/epd2.70295 (PMC13695477; doi:10.1002/epd2.70295)
Supplement: Supplementary file 1 [file EPD2-28-1756-s002.docx]

Answers

1. Correct Answer: B

Rationale: SYNGAP1 absences show a distinctive Jeavons-like posterior onset with crescendo to generalized spike–wave, often preceded by slow eyelid closure.

2. Correct Answer: B

Rationale: Several cohort studies describe slow/disorganized backgrounds, posterior IEDs, and sleep abnormalities linking to developmental severity

3. Correct Answer: C

Rationale: Unlike CAE's brief staring, SYNGAP1 features eyelid myoclonia, atonic components, and reflex triggers like fever/heat.
